# Eine hohe Lebensqualität reduziert depressive Symptome von Maßregelvollzugspatientinnen und -patienten

**DOI:** 10.1007/s11757-022-00732-w

**Published:** 2022-09-21

**Authors:** Ferdinand Bortenschlager, Michael Büsselmann, Jörg Wolstein

**Affiliations:** 1 Institut für Psychologie, Professur für Pathopsychologie, Universität Bamberg, Bamberg, Deutschland; 2 Abteilung für Forensische Psychiatrie und Psychotherapie, Universität Ulm, Ulm, Deutschland

**Keywords:** Forensische Psychiatrie, Unterbringungsqualität, Depression, Versorgungsqualität, MQPL, Forensic psychiatry, Quality of accomodation, Depression, Quality of care, MQPL

## Abstract

**Einleitung:**

Die restriktiven Unterbringungsbedingungen im Maßregelvollzug beeinflussen die Lebensqualität und Depressivität der Untergebrachten und damit die psychische Gesundheit und den Behandlungserfolg therapeutischer Maßnahmen.

**Ziele:**

Ziel der Arbeit ist es, eine Übersicht über die Lebensqualität und die Belastung durch depressive Symptome aus Patientensicht zu erhalten. Es soll analysiert werden, welche Bereiche der Lebensqualität im Zusammenhang mit Depressivität stehen, und ob die Dauer der Maßregel Einfluss auf die Depressivität der Untergebrachten nimmt.

**Methode:**

Die Lebensqualität wurde mittels einer an die Gegebenheiten des Maßregelvollzugs adaptierten Version des Fragebogens Measuring the Quality of Prison Life (MQPL) erhoben. Depressivität wurde anhand der Allgemeinen Depressionsskala (ADS) erfragt. Insgesamt nahmen *N* = 73 Personen an der Studie teil.

**Ergebnisse:**

Die Lebensqualität wurde im Mittel als positiv bewertet. Am höchsten wurde die Beziehung zum therapeutischen Personal eingeschätzt. Es zeigten sich signifikante Unterschiede in der Lebensqualität zwischen depressiv auffälligen und nichtauffälligen Untergebrachten. Die Dauer der Unterbringung war signifikanter Prädiktor für die Depressionswerte suchterkrankter Untergebrachter.

**Fazit:**

Lebensqualität und Depressivität stehen in signifikant negativem Zusammenhang. Zur Verbesserung der Unterbringungs- und Versorgungsqualität sollte das Klinikpersonal die Lebensqualität der Untergebrachten in den identifizierten Bereichen bestmöglich fördern und besonders zum Aufnahmezeitpunkt sowie bei längeren Behandlungsdauern depressive Symptome identifizieren und behandeln. Hierbei sollte ein Augenmerk auf das Erreichen einer stabilen therapeutischen Allianz gelegt werden.

## Abstract (en)

**Introduction:**

The restrictive conditions of placement in forensic psychiatry institutions influence the quality of life and depressiveness of those placed in the hospital and thus the mental health and the success of therapeutic measures.

**Objective:**

The aim of the study is to obtain an insight into the quality of life and the burden of depressive symptoms from the patient’s point of view. It will be analyzed which domains of quality of life are related to depressive symptoms and whether the duration of the treatment has an influence on the depressiveness of the patients.

**Methods:**

Quality of life was assessed using the Measuring the Quality of Prison Life (MQPL) questionnaire, adapted to the conditions of forensic psychiatry. Depressive symptoms were assessed using the General Depression Scale (ADS). A total of *N* = 73 subjects participated in the study.

**Results:**

Quality of life was positively rated on average. The relationship with the therapeutic staff was rated highest. In addition, there were significant differences in quality of life between patients with severe depressive symptoms and those without. Duration of detention was a significant predictor of depression scores of addicted patients.

**Conclusion:**

Quality of life and depressiveness are significantly negatively correlated. To improve the quality of accommodation and care, clinical staff should promote the quality of life of those accommodated in the identified areas to the best of their ability and identify and treat depressive symptoms, especially at the time of admission and during longer treatment periods. In this context, attention should be paid to achieving a stable therapeutic alliance.

## Einleitung

Das Thema „Lebensqualität“ ist bereits seit geraumer Zeit Forschungsgegenstand diverser wissenschaftlicher Unternehmungen und fand im Verlauf der letzten Jahrzehnte vermehrt Eingang in die medizinische sowie forensisch-psychiatrische Forschung (Markiewicz und Wiecek 2017). Trotz dessen besteht in der Literatur keine einheitliche Definition, wobei meist eine Übereinkunft darüber besteht, dass es sich bei Lebensqualität um ein subjektives Konstrukt handelt, welches multidimensional ist und hierin positive wie negative Dimensionen beinhaltet (WHOQOL Group 1995; van Nieuwenhuizen et al. 2002). Aus dieser inhaltlichen Übereinkunft definierte die Weltgesundheitsorganisation (WHO), dass Lebensqualität auf komplexe Art und Weise die körperliche und psychische Gesundheit, das Maß an Unabhängigkeit, die sozialen Beziehungen, persönlichen Überzeugungen und die Umwelt einer Person integriert (WHOQOL Group 1995). Es ist ersichtlich, dass sich bei einer Unterbringung, wie beispielsweise im Justiz- oder im Maßregelvollzug, Einschränkungen in der Lebensqualität ergeben. Um diesen spezifischen Bedingungen von Vollzugseinrichtungen Rechnung zu tragen, sollten Messinstrumente von Lebensqualität den Gegebenheiten der Einrichtungen angepasst werden (van Nieuwenhuizen et al. 2002). Um dies umzusetzen, entwickelte die Forschergruppe um Alison Liebling den Fragebogen* Measuring the Quality of Prison Life* (MQPL; Liebling et al. 2011). Dieser erfasst die Lebensqualität der Gefangenen anhand deren Zufriedenheit mit für den Gefängnisalltag relevanten Domänen wie Respekt, Menschlichkeit, Beziehungen zu Angestellten, Fairness, Vertrauen, Kontakt zu Angehörigen oder Sicherheit (Liebling et al. 2011). Studien konnten die Relevanz von Lebensqualität bzw. einzelnen Domänen unterstreichen: Beijersbergen et al. (2015) konnten in einer niederländischen Gefängnisstichprobe (*n* = 1241) feststellen, dass ein respektvoller Umgang und das Gefühl, fair und gerecht behandelt zu werden, mit einem signifikant niedrigeren delinquenten Rückfallrisiko innerhalb der nächsten 18 Monate einhergingen. Auch konnte ein negativer Zusammenhang zwischen Lebensqualität und Suizidalität bei englischen (Liebling et al. 2005) und US-amerikanischen (Dye 2010) Gefängnispopulationen aufgezeigt werden. In forensischen Stichproben fanden sich Zusammenhänge zwischen Beschränkungen der Lebensqualität und erhöhter Feindseligkeit, Depressivität und Suizidgedanken (Franke et al. 2019). Auch Büsselmann et al. (2020) konnten anhand einer Stichprobe von 159 Maßregelvollzugspatientinnen und -patienten darstellen, dass eine hohe Lebensqualität Depressivität, Hoffnungslosigkeit und Suizidgedanken reduzieren kann. Bouman et al. (2008) postulieren, dass die Erhebung der Lebensqualität zu Beginn der Unterbringung Aufschluss über zu behandelnde Problembereiche geben kann und somit den Behandlungserfolg erhöhen kann. Eben jene erfolgreiche Behandlung ist im Maßregelvollzug zentral, um das Risiko für erneute Delinquenz zu reduzieren und die Maßregel zu beenden (Müller et al. 2017).

Parallel zu den in der Unterbringung inhärenten Einschränkungen in der Lebensführung stellt auch die affektive Verfassung der Untergebrachten einen großen Einflussfaktor auf die Lebensqualität dar (Bullinger et al. 2019), wobei eine zum depressiven Pol verschobene Affektivität negativ mit der Lebensqualität assoziiert ist (Kolovos et al. 2016; Püschner 2012; Renneberg und Lippke 2006). Durch eine Verbesserung der Lebensqualität treten weniger depressive Symptome auf, umgekehrt verbessert sich die Lebensqualität, wenn depressive Symptome reduziert werden (Büsselmann et al. 2020; Franke et al. 2019; Müller et al. 2017; Ruchalla 2017; Sharma et al. 1995). Um eine Belastung durch depressive Symptome zu reduzieren, sollten die Dimensionen der Lebensqualität identifiziert werden, welche die Betroffenen als eingeschränkt bewerten (Büsselmann et al. 2020; Franke et al. 2019; Kolovos et al. 2016; Ruchalla 2017).

### Ziele

Diese Arbeit soll einen weiteren Schritt in der Erforschung der Lebensqualität im Maßregelvollzug darstellen und dieses wenig untersuchte Forschungsfeld bereichern. Hierfür wurden zunächst (1) die Einschätzung der Lebensqualität sowie Depressionswerte in einer Klinik für Forensische Psychiatrie und Psychotherapie erhoben und berichtet.

Um zu identifizieren, welche Bereiche der Lebensqualität für das Auftreten depressiver Symptome besonders relevant sind, und welche Zusammenhänge zwischen Lebensqualität und Depressivität bestehen, wurden (2) Unterschiede in der Lebensqualität zwischen depressiv auffälligen und nichtauffälligen Teilnehmenden untersucht.

Während suchtkranke Untergebrachte in der Regel maximal 2 Jahre im Maßregelvollzug verbleiben, liegt die mittlere Unterbringungsdauer für psychisch kranke Straftäterinnen und Straftäter bei ca. sechseinhalb Jahren (de Tribolet-Hardy und Habermeyer 2016; Dessecker 2008). Dementsprechend befinden sich im Maßregelvollzug Untergebrachte oft lange Zeit in ihren Einrichtungen und sind den einhergehenden Einschränkungen über lange Zeiträume ausgesetzt, weswegen (3) ein Einfluss der Unterbringungsdauer auf die Depressivität untersucht wurde.

Es sollen Implikationen für Forschung und Behandlungspraxis gewonnen werden, um die Lebensbedingungen im Maßregelvollzug an gegebener Stelle zu verbessern und die Belastung durch depressive Symptome zu verringern. Dies kann zur Verbesserung der Versorgungs- und Unterbringungsqualität im Maßregelvollzug beitragen.

## Methode

### Vorgehen

Die Datenerhebung erfolgte zwischen November 2020 und Februar 2021 in einer bayerischen Maßregelvollzugseinrichtung. Es wurde in Kleingruppen über Zweck und Verwendung der Daten, Vorgehen, Messinstrumente, Freiwilligkeit der Teilnahme sowie die fehlende Vergütung aufgeklärt. Die Teilnehmenden gaben eine schriftliche Einverständniserklärung ab und generierten einen individuellen Patientencode, um eine personenbezogene Datenlöschung zu ermöglichen. Hierfür wurden die Kontaktdaten des Versuchsleiters (F.B.) hinterlegt. Die Studiendurchführung erfolgte dementsprechend im Einklang mit der Deklaration von Helsinki. Der Versuchsleiter verblieb während der Erhebung im Raum, um bei Nachfrage Hilfe anbieten zu können. Das Vorgehen war für jede Station der Einrichtung identisch.

### Stichprobe

Eingeschlossen wurden alle volljährigen Patientinnen und Patienten, die nach Auffassung des Behandlungsteams in der Lage waren, die Patienteninformation zu verstehen und die Fragebogen adäquat zu beantworten. Insgesamt nahmen *N* = 73 untergebrachte Personen (weiblich: *n* = 2) an der Befragung teil. Zum Erhebungszeitpunkt befanden sich 264 Untergebrachte in der Einrichtung, woraus sich eine Teilnehmendenquote von 27,7 % ergibt. Aus den Analysen wurden Teilnehmende ausgeschlossen, wenn sie die ADS unvollständig oder einseitig ausgefüllt hatten (*n* = 8) oder den aMQPL unvollständig beantworteten, sodass keine verlässlichen Mittelwerte berechnet werden konnten (*n* = 7). Es ergab sich eine finale Stichprobe von *n* = 58. Nach § 63 StGB waren *n* = 7 (12,1 %) der Teilnehmenden untergebracht, *n* = 51 (87,9 %) nach § 64 StGB. Das Durchschnittsalter betrug etwa 35,5 Jahre (Range: 20–71; *SD* = ± 11,00). Drei Untergebrachte (5,2 %) gaben an, an einer depressiven Erkrankung zu leiden. Für einen genaueren Überblick über die Merkmale der Stichprobe: Tab. 1.

**Table 1:** 

		Teilnehmende (*n* = 58) *M* (*SD*; Range) Anzahl (%)
*Unterbringungsgrundlage*	§ 63 StGB	7 (12,1 %)
	§ 64 StGB	51 (87,9 %)
*Alter (in Jahren)*		35,5 (11,00; 20–71)
*Schulabschluss*	Kein Abschluss	11 (19,0 %)
	Haupt‑/Mittelschulabschluss	32 (55,2 %)
	Realschulabschluss	10 (17,2 %)
	(Fach‑)Abitur	5 (8,6 %)
*Hauptdiagnose*^*a*^	Suchterkrankung	45 (77,6 %)
	Schizophrenie	2 (3,4 %)
	Sonstige (z. B. Paraphilien)	4 (6,9 %)
	Patienten mit Mehrfachdiagnose	10 (17,2 %)
*Schwerstes Anlassdelikt*^*b*^	Tötungsdelikt	1 (1,7 %)
	Raub	3 (5,2 %)
	Körperverletzung	10 (17,2 %)
	Diebstahl	7 (12,1 %)
	Betrug	1 (1,7 %)
	Verstoß gegen das BtMG	25 (43,1 %)
	Sexualdelikt	3 (5,2 %)
	Sonstiges (z. B. Verkehrsdelikt)	3 (5,2 %)
*Behandlungsdauer (in Monaten)*	Für Patienten nach § 64 StGB	8,7 (9,28; 1–48)
	Für Patienten nach § 63 StGB	114,7 (139,37; 24–417)
	Gesamt	21,5 (57,74; 1–417)

^a^Fehlende Angaben: *n* = 7

^b^Fehlende Angaben: *n* = 5

### Materialien

Für die Erfassung demografischer, forensisch-klinischer sowie juristischer Daten wurde ein Kurzfragebogen erstellt. Dieser wurde vor der Erfassung der Lebensqualität sowie der Depressionsskala ausgefüllt und beinhaltete Fragen zu Alter, Geschlecht, Schulabschluss, Unterbringungsdauer, Abbruchstatus, Diagnosen, Einweisungsdelikt, Lockerungsstufe und Unterbringungsparagraph. Aufbau und Struktur des Fragebogens waren an Büsselmann et al. (2021) angelehnt

Die Lebensqualität wurde durch eine von Büsselmann et al. (2021) aus dem Englischen übersetzte und an die Gegebenheiten des Maßregelvollzugs adaptierte Version des MQPL (Liebling et al. 2011) erfasst. Items zu therapeutischer Hilfe und Unterstützung im Gefängnis wurden entfernt und durch Items aus dem Fragebogen zur therapeutischen Beziehung in der Forensik (FTBF; Vasic et al. 2015) ersetzt. Der aus der Adaptation resultierende Fragebogen *aMQPL* besteht aus 64 Items auf 11 Skalen. Die Reliabilität des aMQPL kann als exzellent bewertet werden (Cronbachs Alpha des Gesamtwertes: *r* = 0,951).

Depressive Symptome wurden mit der Allgemeinen Depressionsskala (*ADS*) von Hautzinger und Bailer (1993) erhoben. Die ADS erfasst die Beeinträchtigung durch depressive Symptome innerhalb der letzten 7 Tage mittels 20 Items. Durch die Berechnung von Summenwerten sowie die Angabe eines Cut-off-Wertes sind sowohl kategoriale als auch dimensionale Auswertungen möglich. Die Autoren nannten einen kritischen Summenwert von 22 Punkten, nach dessen Überschreiten Teilnehmende als depressiv auffällig gelten (Hautzinger et al. 2012; Hautzinger und Bailer 1993).

### Statistische Analysen

Um die erste Forschungsfrage zu beantworten, wurden für Kurzfragebogen, aMQPL und ADS deskriptive Statistiken berechnet. Fünf Teilnehmende hatten nur 19 der 20 ADS-Items beantwortet. Für diese wurde der fehlende Wert durch den Mittelwert der gültigen Antworten ersetzt (Hautzinger et al. 2012). Daraufhin wurden für die ADS-Summenwerte berechnet und in T‑Werte transformiert.

Zur Untersuchung der zweiten Forschungsfrage wurde eine binäre Variable für ADS-Summenwerte über bzw. unter 22 Punkten erstellt. Für die statistische Analyse von Mittelwertsunterschieden in der Lebensqualität wurde auf generalisierte lineare Modelle zurückgegriffen, welche nicht den Anforderungen des allgemeinen linearen Modells unterliegen (Baltes-Götz 2016). Für jede der aMQPL-Subskalen wurde ein generalisiertes binär-logistisches Regressionsmodell berechnet.

Für die Untersuchung der dritten Forschungsfrage wurden nur nach § 64 StGB Untergebrachte betrachtet (*n* = 51), da die deutlich längeren Aufenthaltsdauern der nach § 63 StGB Untergebrachten die Zusammenhänge stark verzerren würden. Es wurde eine Kurvenanpassung berechnet, um das Zusammenhangsmodell mit der höchsten Varianzaufklärung in den Depressionswerten zu identifizieren. Durch die Verwendung von ADS-T-Werten konnten Einflüsse des Alters und des Geschlechts ausgeschlossen werden.

Auch wurde ein Mann-Whitney-U-Test für unabhängige Stichproben durchgeführt, um Unterschiede zwischen den Patientengruppen, aufgeteilt nach den Unterbringungsparagraphen, zu analysieren.

Alle oben genannten statistischen Analysen wurden mittels IBM SPSS Statistics für Windows, Version 27 (IBM Corp., Armonk, NY, USA) berechnet.

## Ergebnisse

Der aMQPL-Gesamtwert lag mit 2,43 (*SD* = 0,54) Punkten im positiven Bereich (Tab. 2). Am höchsten wurde die Beziehung zum therapeutischen Personal bewertet (*M* = 3,16; *SD* = 0,76). Am unzufriedensten waren die Untergebrachten mit dem Kontakt zu ihren Angehörigen (*M* = 1,86; *SD* = 1,04).

**Table 2:** 

aMQPL-Skala	Mittelwert (*SD*)
Aufnahme in den Maßregelvollzug	2,44 (0,76)
Beziehung zu Mitpatienten	2,41 (0,77)
Beziehung zu Pflegekräften	2,61 (0,90)
Beziehung zu Therapeuten	3,16 (0,76)
Kontakt zu Angehörigen	1,86 (1,04)
Respektvoller Umgang	2,58 (0,76)
Gleichbehandlung der Patienten	2,09 (1,04)
Transparenz der Abläufe und Entscheidungen	2,33 (0,75)
Sicherheitserleben	2,46 (0,80)
Qualität der Unterbringung	2,37 (0,55)
Therapeutische Angebote	2,42 (0,33)
Gesamt	2,43 (0,54)

*n* = 58

**Figure 1:**
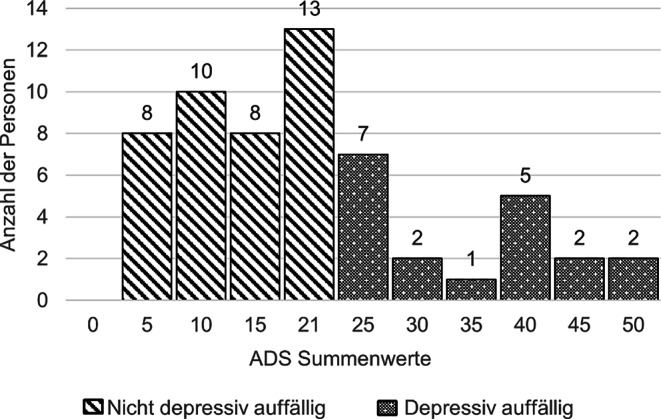

Der Mittelwert der ADS-Summen lag bei 18,48 (*SD* = 12,37) Punkten. Über dem Cut-off-Wert von 22 Punkten lagen *n* = 19 (32,8 %) Teilnehmende und wurden als depressiv auffällig kategorisiert (Abb. 1).

Zudem ergaben sich signifikante Unterschiede zwischen den Untergebrachten nach §§ 63 und 64 StGB in Alter und Behandlungsdauer (jeweils *p* < 0,05). § 63-StGB-Patienten waren im Schnitt älter und länger untergebracht. Der aMQPL-Gesamtwert sowie die ADS-Summenwerte unterschieden sich nicht signifikant zwischen den Gruppen.

### Unterschiede in der Lebensqualität zwischen depressiv auffälligen und nicht depressiv auffälligen Teilnehmenden

**Figure 2:**
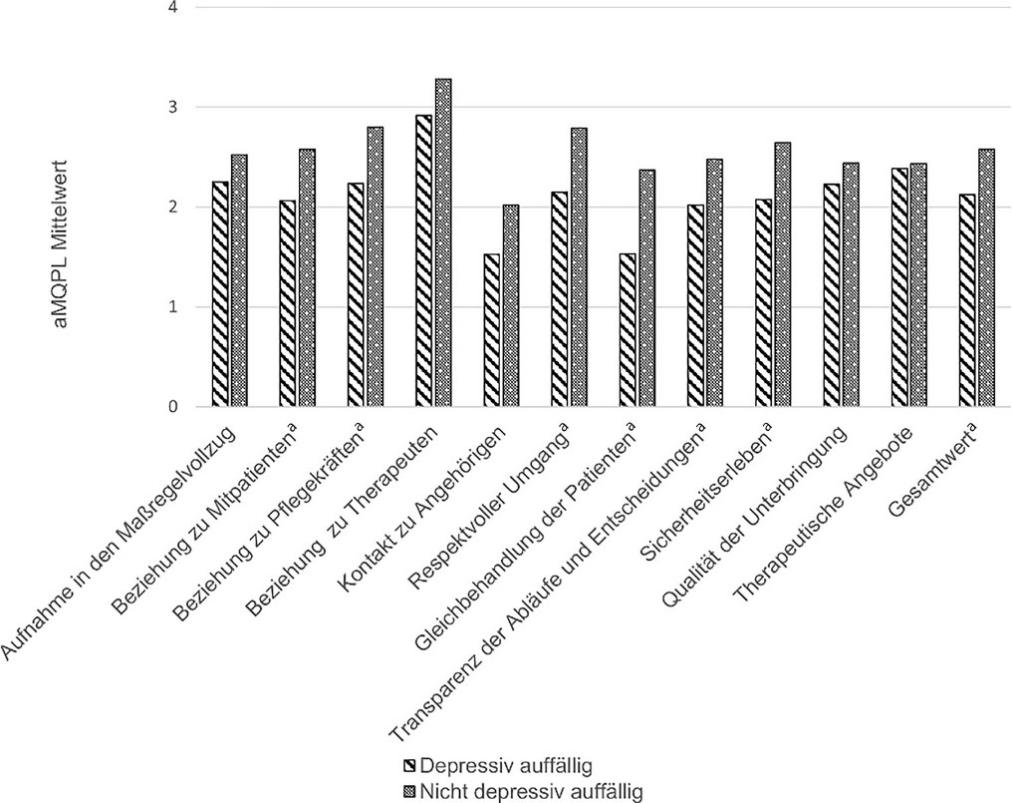

Insgesamt berichten depressiv auffällige Teilnehmende von einer signifikant niedrigeren Lebensqualität (*M*_*auffällig_gesamt*_ = 2,13; *SD* = 0,439) als nichtauffällige (*M*_nicht_auffällig_gesamt_ = 2,58; *SD* = 0,523, Abb. 2). Analog zur Gesamtstichprobe wurde der Kontakt zu Angehörigen am niedrigsten bewertet (*M* = 1,53; *SD* = 0,918) und der Kontakt zu dem therapeutischen Team am höchsten (*M* = 2,92; *SD* = 0,832).

Die Skalen *Beziehung zu Mitpatienten, respektvoller Umgang* und *Sicherheitserleben* hatten signifikante Effekte auf die Depressivität. Ein großer Effekt zeigte sich für den Gesamtwert der aMQPL-Skala (Cohen’s *d* = −1,19; Cohen 1988). Je höher die Lebensqualität ausgeprägt war, desto geringer war das Risiko für depressive Auffälligkeit und vice versa. Eine genauere Darstellung der Unterschiede in der Lebensqualität von depressiv Auffälligen und Nichtauffälligen findet sich in Tab. 3.

**Table 3:** 

Skala	*b*	*p*	Exp (*b*)	95 %-KI für Exp (*b*)		*d*^a^
				Unterer Wert	Oberer Wert	
Aufnahme in den Maßregelvollzug	−0,478	0,199	0,605	0,299	1,286	−0,28
Beziehung zu Mitpatienten	−0,937*	0,022	0,392	0,176	0,871	−0,52
Beziehung zu Pflegekräften	−0,713*	0,032	0,490	0,256	0,939	−0,39
Beziehung zu Therapeuten	−0,636	0,093	0,529	0,252	1,112	−0,35
Kontakt zu Angehörigen	−0,563	0,075	0,570	0,306	1,059	−0,31
Respektvoller Umgang	−1,281*	0,004	0,278	0,115	0,669	−0,71
Gleichbehandlung der Patienten	−0,879*	0,006	0,415	0,222	0,777	−0,48
Transparenz der Abläufe und Entscheidungen	−0,881*	0,033	0,415	0,184	0,932	−0,48
Sicherheitserleben	−0,961*	0,016	0,382	0,175	0,834	−0,53
Qualität der Unterbringung	−0,723	0,168	0,485	0,174	1,356	−0,40
Therapeutische Angebote	−0,477	0,573	0,621	0,118	3,260	−0,26
Gesamtwert	−1,788*	0,005	0,116	0,049	0,575	−1,19

*Signifikanter Wert bei Signifikanzniveau α = 0,05

^a^Interpretation von Cohen’s *d*: großer Effekt = |0,80|, mittlerer Effekt = |0,50|, kleiner Effekt = |0,20|

### Depressionswerte in Abhängigkeit von der Unterbringungsdauer

**Figure 3:**
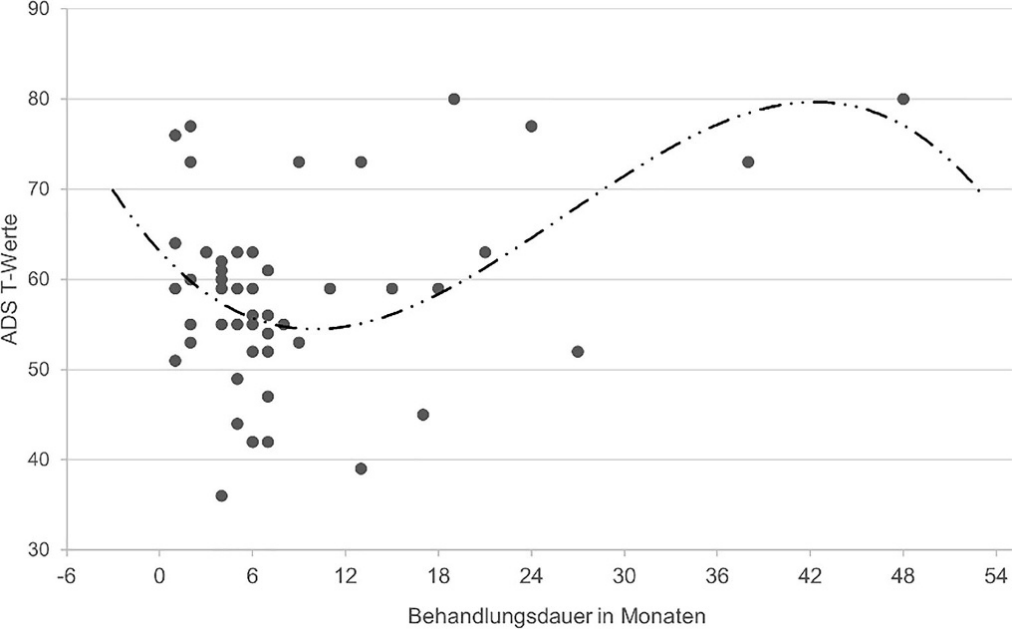

Wie in Abb. 3 dargestellt, ergab die Berechnung einer Kurvenanpassung ein Regressionsmodell mit kubischem Zusammenhang zwischen Behandlungsdauern und ADS-T-Werten, welches 20,9 % der Varianz in den Depressionswerten aufklären konnte. Nach anfänglicher Reduktion stiegen die ADS-Werte ab dem 10. Unterbringungsmonat an, bevor sie wieder abfielen.

## Diskussion

Die Teilnehmenden bewerten ihre Lebensqualität als positiv. Dieser Befund deckt sich mit vorherigen Studien, welche die Lebensqualität im Maßregelvollzug mittels des aMQPL analysierten (Büsselmann et al. 2020; Franke et al. 2019). Bis auf den Kontakt zu Angehörigen wurden alle Skalen tendenziell als positiv (Mittelwerte > 2,0) bewertet. Dementsprechend ist davon auszugehen, dass seitens der klinischen Einrichtung kein unmittelbarer Aufwand zur Verbesserung der Lebensbedingungen betrieben werden muss, um die Versorgungsqualität zu erhöhen. Der Kontakt zu Angehörigen könnte je nach Personalsituation durch vermehrte Ausgänge oder das Schaffen alternativer Kontaktmöglichkeiten wie Videotelefonie verbessert werden. Das Niveau der Lebensqualität sollte aufrechterhalten werden.

Die Beziehung zum therapeutischen Personal wurde am höchsten bewertet. Im Maßregelvollzug Untergebrachte verbleiben oftmals viele Monate bis Jahre in der Einrichtung, sodass zum Aufbau einer stabilen, tragfähigen Beziehung zwischen Patientin oder Patient und Therapeutin bzw. Therapeut viel Zeit zur Verfügung steht (Otte et al. 2019). Die therapeutische Beziehung stellt einen wichtigen Faktor im Behandlungserfolg dar und beeinflusst u. a. die Symptomreduktion, Medikamentenadhärenz und Reinstitutionalsisierungswahrscheinlichkeit (Höfer et al. 2015). Ebenso besteht ein Zusammenhang zwischen der Beziehungsqualität und der Richtigkeit des Assessments des Gewaltrisikos der Patienten (Höfer et al. 2015).

Der Mittelwert der ADS-Antworten lag mit 18,48 (*SD* = 12,37) Punkten mit über 5 Punkten um ca. eine halbe Standardabweichung über dem Normmittelwert (*M* = 13,2; *SD* = 9,6) (Hautzinger et al. 2012). Während *n* = 19 (32,8 %) der Teilnehmenden als depressiv auffällig kategorisiert wurden, lag lediglich bei *n* = 3 (5,2 %) Personen eine dementsprechende Diagnose vor. Depressive Erkrankungen resp. die Belastung durch depressive Symptome werden im Maßregelvollzug evtl. unterschätzt. Dies ist für einen erfolgreichen Therapieverlauf kritisch zu betrachten, da sich eine komorbide depressive Erkrankung in der Regel negativ auf den Verlauf der Primärerkrankung und den Therapieprozess auswirkt (DGPPN et al. 2017; DiMatteo et al. 2000; Katon und Sullivan 1990; Sharma et al. 1995). Da depressive Symptome die Entwicklung weiterer psychischer Krankheiten begünstigen und das Suizidrisiko erhöhen, sollten Patientinnen und Patienten des Maßregelvollzuges im Hinblick auf eine optimale Versorgung auf depressive Symptome gescreent und, wenn diese vorliegen, behandelt werden (Härter et al. 2018; Wolfersdorfer et al. 2016). Auch sind depressive Erkrankungen in Bezug auf eine positive Legalprognose als Zielkriterium einer Maßregel als negative Einflussfaktoren zu betrachten. Fazel et al. (2015) konnten anhand einer schwedischen Population depressiv erkrankter Personen (*n* = 47.158) ein 3-fach erhöhtes Risiko für Gewaltstraftaten im Vergleich zur Normalbevölkerung (*n* = 898.454) identifizieren. Eine komorbide depressive Erkrankung kann dazu führen, dass die Zufriedenheit mit den aktuellen Lebensbedingungen grundsätzlich als geringer eingeschätzt wird (Ellert et al. 2005). In diesem Falle obliegt es dem Klinikpersonal, die Depression zu behandeln, um die Lebensqualität der Patientinnen und Patienten zu steigern. Umgekehrt kann eine geringe Lebensqualität zu depressiven Symptomen führen. Hier sollte das Behandlungsteam die Problembereiche in der Lebens- und Versorgungsqualität der Patientinnen und Patienten ansprechen und durch eine Veränderung dieser Einschränkungen die Lebensqualität erhöhen.

Zwischen den ADS-T-Werten und der Unterbringungsdauer konnte ein kubischer Zusammenhang identifiziert werden. Die höheren Depressionswerte zu Beginn der Unterbringung können u. a. dadurch erklärt werden, dass eine Inhaftierung und Verhandlung samt Verurteilung einschneidende Erlebnisse darstellen, welche psychische Belastungen verursachen und dadurch depressive Symptome begünstigen können (Affandi et al. 2018). Zudem bestehen bei Abhängigkeitserkrankungen hohe Komorbiditäten mit depressiven Erkrankungen; Schäfer und Heinz (2005) berichten von Lebenszeitprävalenzen von depressiven Störungen bei alkoholabhängigen Patientinnen und Patienten zwischen 54 und 75 %, bei opiatabhängigen zwischen 38 und 56 %. In den hohen ADS-Werten zu Beginn der Unterbringung spiegelt sich evtl. diese Komorbidität.

## Limitationen und Implikationen

Die Stichprobengröße ist eine Limitation dieser Studie. Insbesondere war der Anteil psychisch erkrankter Patientinnen und Patienten nach § 63 StGB mit *n* = 7 gering.

Weiter mussten 15 Datensätze aufgrund unvollständiger oder fehlerhafter Beantwortungen aus der Analyse ausgeschlossen werden, was die Aussagekraft der Ergebnisse schmälert.

Die Daten wurden an einem Standort erhoben und können evtl. nur eingeschränkt auf andere Maßregelvollzugskliniken übertragen werden. Besonders gilt es, die identifizierten Phasen der gesteigerten Belastung durch depressive Symptome weiter zu untersuchen und zu überprüfen, ob es sich hierbei um ein verallgemeinerbares oder klinikspezifisches Ergebnis handelt.

Aufgrund der methodischen Gegebenheiten dieser Studie sind keine kausalen Interpretationen der Ergebnisse zulässig. Zukünftige Forschungsarbeiten sollten Designs umsetzten, die kausale Interpretationen ermöglichen.

Die Bewertung des Kontakts zu Angehörigen, aber auch die anderen Skalen des aMQPL sowie die Antworten auf der ADS unterliegen dem Einfluss der aktuell vorherrschenden COVID-19-Pandemie. Bäuerle et al. (2020) konnten beispielsweise eine Zunahme an depressiven Symptomen während der Pandemie in der Normalbevölkerung feststellen. Im Setting der Forensischen Psychiatrie könnten ähnliche Effekte verzerrende Auswirkungen auf die Erfassung der Lebensqualität sowie Depressivität gehabt haben. Dementsprechend sollten Einflüsse der Coronapandemie auf die Lebensqualität und die psychische Verfassung der Untergebrachten analysiert werden, um temporäre pandemische Einflüsse von zeitlich stabilen Qualitäten der Einrichtungen und anderen Faktoren auf die Lebensqualität unterscheiden zu können. Auch kann der aMQPL als Instrument zur Verlaufsmessung Veränderungen in den Lebensbedingungen der Untergebrachten identifizieren und beispielsweise die therapeutische Allianz im Sinne des Qualitätsmanagements regelmäßig überprüfen.

## Fazit

Lebensqualität und Depressivität stehen in signifikant negativem Zusammenhang. Zur Verbesserung der Unterbringungs- und Versorgungsqualität sollten die Einrichtungen die Lebensqualität der Untergebrachten in den identifizierten Bereichen bestmöglich fördern und besonders zum Aufnahmezeitpunkt sowie bei längeren Behandlungsdauern ab dem 10. Unterbringungsmonat depressive Symptome identifizieren und behandeln. Ein weiteres Augenmerk sollte auf die therapeutische Beziehung gelegt werden, da diese einen positiven Effekt auf den gesamten Behandlungserfolg bedeutet. Auf eine hohe Lebensqualität und eine geringe Belastung durch depressive Symptome zu achten, kann zu einer Verbesserung der Versorgungs- und Unterbringungsqualität der jeweiligen Einrichtungen beitragen. Zukünftige Studien sollten größere Stichproben beinhalten und Designs realisieren, welche kausale Interpretationen zulassen.
